# Fathead minnow steroidogenesis: *in silico *analyses reveals tradeoffs between nominal target efficacy and robustness to cross-talk

**DOI:** 10.1186/1752-0509-4-89

**Published:** 2010-06-28

**Authors:** Jason E Shoemaker, Kalyan Gayen, Natàlia Garcia-Reyero, Edward J Perkins, Daniel L Villeneuve, Li Liu, Francis J Doyle

**Affiliations:** 1 Dept of Chemical Engineering, University of California, Santa Barbara, Santa Barbara, CA, USA; 2 Dept of Chemistry, Jackson State University, Jackson, MS USA; 3 U.S. Army Engineering Research and Development Center, 3909 Halls Ferry Road, Vicksburg, Mississippi USA; 4 U.S. EPA Mid-Continent Ecology Division, 6201 Congdon Blvd., Duluth, MN USA; 5 Interdisciplinary Center for Biotechnology Research, University of Florida, Gainesville, FL, USA

## Abstract

**Background:**

Interpreting proteomic and genomic data is a major challenge in predictive ecotoxicology that can be addressed by a systems biology approach. Mathematical modeling provides an organizational platform to consolidate protein dynamics with possible genomic regulation. Here, a model of ovarian steroidogenesis in the fathead minnow, *Pimephales promelas*, (FHM) is developed to evaluate possible transcriptional regulation of steroid production observed in microarray studies.

**Results:**

The model was developed from literature sources, integrating key signaling components (G-protein and PKA activation) with their ensuing effect on steroid production. The model properly predicted trajectory behavior of estradiol and testosterone when fish were exposed to fadrozole, a specific aromatase inhibitor, but failed to predict the steroid hormone behavior occurring one week post-exposure as well as the increase in steroid levels when the stressor was removed. In vivo microarray data implicated three modes of regulation which may account for over-production of steroids during a depuration phase (when the stressor is removed): P450 enzyme up-regulation, inhibin down-regulation, and luteinizing hormone receptor up-regulation. Simulation studies and sensitivity analysis were used to evaluate each case as possible source of compensation to endocrine stress.

**Conclusions:**

Simulation studies of the testosterone and estradiol response to regulation observed in microarray data supported the hypothesis that the FHM steroidogenesis network compensated for endocrine stress by modulating the sensitivity of the ovarian network to global cues coming from the hypothalamus and pituitary. Model predictions of luteinizing hormone receptor regulation were consistent with depuration and in vitro data. These results challenge the traditional approach to network elucidation in systems biology. Generally, the most sensitive interactions in a network are targeted for further elucidation but microarray evidence shows that homeostatic regulation of the steroidogenic network is likely maintained by a mildly sensitive interaction. We hypothesize that effective network elucidation must consider both the sensitivity of the target as well as the target's robustness to biological noise (in this case, to cross-talk) when identifying possible points of regulation.

## Background

Recently, the field of toxicology has begun to shift from an observational study of disease-specific models in vivo to a more predictive science focusing on mechanism-based, biological observations in vitro using high throughput technologies [1]. This transition was prompted by the increasingly large number of substances needing to be tested, the need to better relate to human and animal data, and the overall expense of disease-specific studies [1-3]. A specific challenge in toxicology is the ability to identify or predict compensatory responses that occur in response to trace levels of poison exposure [4]. These challenges are best addressed using systems approaches which focus on organizing and exploring the complex networks affected during toxin exposure and response. High-throughput technologies such as microarrays can observe the global response of the genome under various conditions, but several challenges remain in addressing causality and data consolidation [5]. Proteomic and genomic behaviors can differ between in vitro and in vivo data sets as inter-organ regulation is removed. Mathematical models provide organizational platforms to generate hypotheses that allow for consistency while interpreting these heterogeneous data sets.

Aspects of biological networks, such as robustness, can be exploited to guide network elucidation [6,7] and reveal pathways critical to the body's response to toxin exposure. Robustness, the ability of a biological network to maintain performance under variable environmental conditions, is an emergent system property often employed to guide model development/reduction [8]. Robustness can be observed at many levels of complexity. Considering viability as a performance measure, mutation studies in yeast [9], flies [10], and mice [11] find that these organisms are robust to single gene deletions in 80-90% of the genome. Robustness properties have been explored in several systems, such as circadian gene regulatory networks [12], chemotaxis [13], and apoptosis [14,15]. For systems described by ordinary differential equations, sensitivity analysis can identify interactions or species (i.e., genes or proteins) which most strongly dictate the behavior of the network output [16], ultimately guiding the next iteration of experimentation and model development.

Sensitivity is the network response to infinitesimal disturbances in either a parameter value or initial condition. It is a dynamic measure (evaluated over time) which can identify optimal experimental conditions and guide model reduction [17]. Bentele *et al. *applied sensitivity analysis to reduce system complexity in a CD95 induced apoptosis model [14]. Applied to circadian rhythm models, sensitivity analysis shows that circadian systems are often more fragile to perturbations in global parameters (transcriptional and translational machinery) than local parameters, a characteristic which appears to be the result of network topology as opposed to parameter tuning [12,18]. Sensitivity analysis is often embedded in design of experiment schemes to identify network interactions which best manipulate the observable outputs [19].

**Figure 1:**
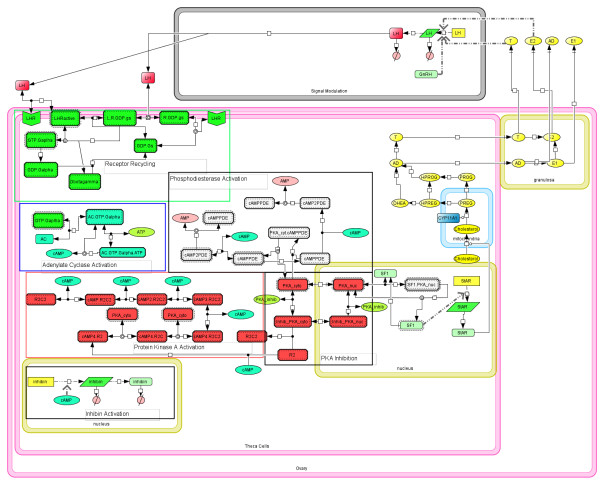
**Model of sex steroid production in ovaries**. Cholesterol transport within ovarian cells is modulated by the feedback of E_2 _and T on LH production in the brain and pituitary. These effects are summarized by the Signal Modulation compartment of the model. When LH binds its receptor, it activates G protein recycling and the activation of adenylate cyclase. This produces the necessary cAMP for PKA and phosphodiesterase activation. The signaling cascade ends at the activation of StAR which allows for the translocation of cholesterol into the mitochondria. Once in the mitochondria, cholesterol goes through a series of enzyme-mediated reactions to produce sex steroids.

In this work, a model of ovarian steroidogenesis in the fathead minnow (*Pimephales promelas*, FHM) is developed to consolidate mRNA and protein data. Steroidogenesis, the production of hormones from cholesterol, is essential to a wide range of physiological and pathological processes [20]. Hormones are powerful physiological regulators, allowing organs to induce changes in distant tissues within the organism. Testosterone (T) and estrogens, such as estrone (E_1_) and estradiol (E_2_), serve as growth hormones for reproductive tissues whose regulation is essential to reproduction as well as several other physiological factors such as bone structure [21] and arterial blood flow [22]. On the pathological side, endocrine inhibiting therapies such as fadrozole (FAD), a specific aromatase inhibitor, are commonly used to slow the progression of estrogen-dependent breast cancers [23]. These hormones are primarily produced in the gonads through a series of enzyme-mediated reactions [24]. Cholesterol availability is regulated by gonadotropin-releasing hormone (GnRH) and luteinizing hormone (LH), which are released from hypothalamic and pituitary tissues, respectively and activate a series of signaling events, ultimately resulting in the production of StAR protein [25,26]. StAR protein facilitates the translocation of cholesterol in the theca cells of the ovary from the outer to the inner mitochondrial membrane, the first step in steroidogenesis (See Figure 1) [27].

Hormone regulation is highly sensitive to endocrine-disrupting compounds (EDCs) released into the environment [4]. EDCs are an environmental health concern as they are potentially hazardous at trace concentrations. The industrial chemical bisphenol A (BPA), used to manufacture polycarbonate plastics, may leach from disposable plastic bottles. Leached BPA can act as an estrogen receptor modulator linked to a variety of issues during early development [28]. Ketoconazole, found in antifungal ointments, and ethinyl estradiol, the active ingredient in most oral birth control pills, both inhibit different aspects of the enzyme machinery essential to the conversion of cholesterol to steroids [29,30]. Since they also affect systems involved in development, exposure effects may not be immediately apparent until many years post-exposure [4]. Mathematical models that can assist in identifying genomic and metabolic signatures for EDC exposures, as well as predicting the effects of EDCs would be especially valuable for understanding the health risk of chemicals in the environment.

The FHM is widely used by the US Environmental Protection Agency as an ecological test organism due to its wide distribution throughout North America, availability, and relatively short generation time [31]. Watanabe *et al*. [32] developed a physiologically-based computational model of steroidogenesis in the male FHM to describe the effects of E_2 _and ethinyl estradiol (EE_2_) exposure. While the model accurately described the impacts of these two chemicals on steroid production, it did not incorporate many of the key signaling pathways that might influence the response of FHM to a variety of EDCs.

To better understand the regulatory response of the steroidogenesis pathway to EDC exposure, a signaling model composed of ordinary differential equations was created to predict the effects of exposure to the aromatase inhibitor fadrozole (FAD) on FHM steroidogenesis. We incorporated into the model key signaling pathways that were suggested by microarray analysis as well as the literature. As the steroidogenesis pathway is considered highly conserved among a variety of species [33], the model of ovarian steroidogenesis was constructed by integrating several mouse and fish models currently available in the literature. We used microarray data from an in vivo and in vitro exposure to FAD to detect potential key signaling pathways involved in steroidogenesis, such as G-protein signaling, kinase activity, and mitochondrial transport. A graphical FHM steroidogenesis model [25] was combined with G protein cycling and protein kinase A (PKA) activation models [34]. These models quantify the intra-ovary signaling which regulates steroidogenic acute regulatory (StAR) gene activation, which in turn regulates the transport of cholesterol into the inner mitochondrial membrane [27]. Once in the mitochondria, steroid production is described by a model of the enzyme-mediated conversion of cholesterol to its steroid derivatives [24]. Extra-ovary signaling occurring along the hypothalamus-pituitary-gonadal (HPG) axis was incorporated using the regulatory effects of E _2 _and T on the production of luteinizing hormone (LH) described by Watanabe *et al*. [32]. We tested our model using data from a new exposure. Fish were exposed to two different concentrations of FAD for eight days, then the chemical was removed and samples were taken for eight more days. Steroid levels were measured. Our model was able to predict the steroid response to the FAD but failed to capture the apparent compensation (in terms of T and E _2 _serum levels) occurring during 8 days exposure as well as the over compensation observed during the depuration phase. Sensitivity analysis and microarray data identified possible missing regulation steps that could be responsible for the over production of these steroids during depuration.

## Results

### Steroidogenesis model development

To consolidate the steroidogenic response between in vivo and in vitro steroid measurements, a G protein signaling model [34] and a model of intra-mitochondrial, enzyme-mediated steroidogenesis [24] were coupled with a mathematical description of LH production. Microarray data from FAD exposures identified mitochondrial transport and G-protein signaling as enriched Gene Ontology (GO) terms. Microarrays also identified many differentially expressed genes related to kinase activity and adenylate cyclase both in the in vivo and in vitro exposure. Due to their close relation to steroidogenesis confirmed by the microarrays, the pathways were incorporated into the model.

LH hormone, produced in the pituitary, is regulated by gonadotropin-releasing hormone (GnRH) released from the hypothalamus [35]. GnRH is released in a pulsatile fashion and the frequency is dependent on several physiological factors [36]. While GnRH dynamics lead to pulsatile LH levels, the magnitude of the oscillations is thoroughly damped by the G-protein signaling process.

LH binds to its receptor, initiating the first step in the G-protein cycle (Figure 1). The original model of G protein cycling, and its ensuing activation of adenylate cyclase/protein kinase A production by Hao *et al*. described the binding of vasoactive intestinal polypeptide (VIP) and CREB activation. While receptor/ligand binding rates may differ between the VIP system and steroidogenesis, the two systems share the same signaling architecture, namely adenylate synthetase (AD) activation, protein kinase A (PKA) activation and phosphodiesterase activation. Thus, the description applied to VIP concentration/CREB activation is applied to LH/SF1 activation (See the Methods section for greater detail on individual reaction steps).

The activation of SF1 promotes the transcription of StAR. The StAR protein is the primary transporter of cholesterol across the mitochondrial membrane, the rate-limiting step of steroid production [37]. Once in the inner mitochondrial membrane, cholesterol is transformed by a series of enzyme-catalyzed reactions, primarily from the cytochrome P450 oxidase enzyme family. Breen *et al*. [24] first modeled the steps between cholesterol and T/E _2 _production, and applied the model to describe steroid production in ovary explants exposed to FAD. Four steroids are produced (androstenedione (AD) and estrone (E_1_) are produced as well), but E_2 _and T are known to inhibit the transcription of LH. Detailed models of the pituitary and hypothalamus are unavailable, thus LH transcription and translation were described by a set of reactions labeled as Signal Modulation (See Figure 1). This strategy reduces the complexity in both the visualization and mathematical modeling of steroidogenesis while still capturing the feedback regulation of steroid production on LH levels.

### Parameter Training

**Figure 2:**
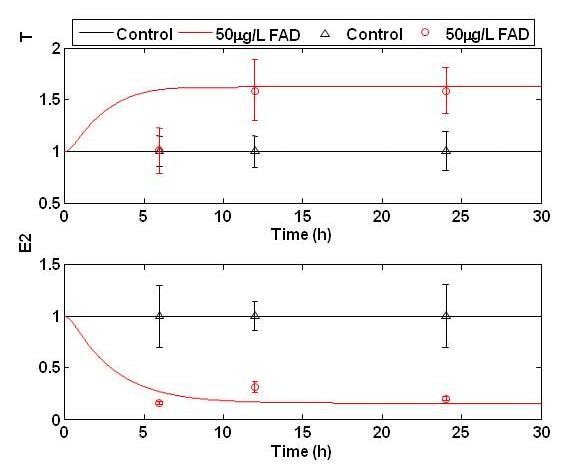
**The fitted model trajectories of E_2 _and T concentration of during to 50 μg FAD/L (red) *ex vivo***. FAD was introduced into the media, and the fish were exposed for 6, 12, and 24 hours. At each time point, fish were removed from the media and ex vivo production of T and E_2 _ovaries measured. Upper triangles represent control measurements while open circles represent exposure measurements. Model predictions are shown in solid black and red lines for control and FAD exposed data, respectively.

While the majority of parameters have been justified in their original publications, measurements are unavailable for parameters related to LH transcription and its inhibition. These parameters were used to train the system to a training data set, and the resulting model was evaluated for both predictive capacity and sensitivity to parameter uncertainty. Given the small number of parameters which required fitting, parameters were operator tuned. Figure 2 shows the fitted response of the steroidogenesis model to T and E_2 _measurements taken from ovaries (ex vivo) stressed with 50 g FAD/L for 6, 12, and 24 h. The model performed well with the exception of the 6 h time point where production of T is overestimated.

### Sensitivity Analysis

**Figure 3:**
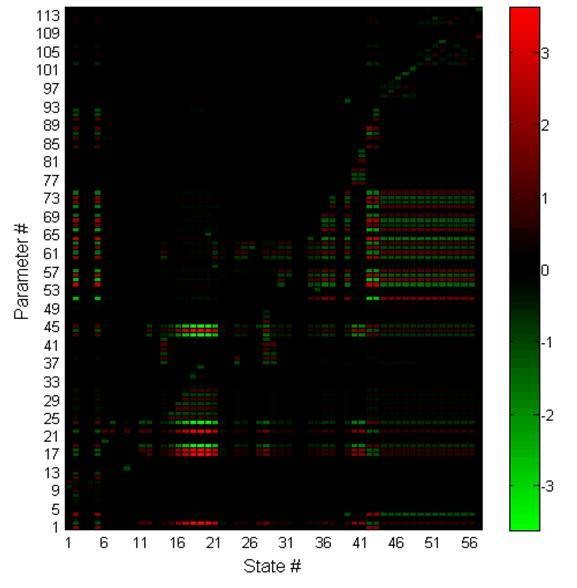
**The relative sensitivity of each state to each parameter, averaged over time**. The sensitivity was calculated over a span of 30 days, and color bars represent the value of the relative sensitivity for each state to each parameter.

To evaluate the sensitivity of the resulting model fitting, the relative sensitivity of the steroidogenesis model was calculated for each state to each parameter, averaged over time (Figure 3). The parameters clustered into various ranges of sensitivity. The most sensitive parameters was generally in the PKA activation cycle (states 16 - 21) and included some interactions outside of the PKA cycle itself, such as basal G-protein activation (parameter 22) and AC activation (parameters 17 - 19). Cholesterol and steroid states (states 39, 44-56) were most sensitive to parameters 54 - 57, 60 - 64, and 71 - 74, which corresponded to PKA inhibition, PKA translocation and the rate of protein synthesis, respectively.

**Figure 4:**
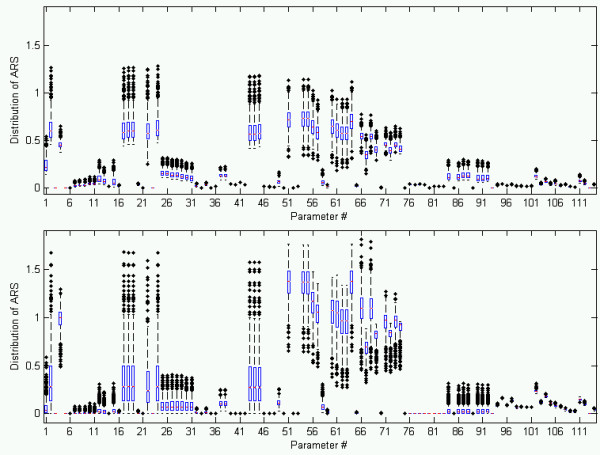
**The average relative sensitivity (ARS) for each parameter and state was calculated over time for 1000 parameter generations**. Sensitivity was then averaged over all states and time for each parameter and its boxplot is shown to illustrate the sensitivity distribution.

We perturbed the nominal parameter set over 1000 parameter generations to determine the robustness of the sensitivity analysis conclusions to the nominal parameter values selected (Figure 4). When all states were considered equal (the relative sensitivity of all states was averaged together), the sensitivity results above remained true. It can be stated with confidence that the steroidogenesis network is very sensitive to noise in the G-protein and PKA activation cycles. Focusing specifically on the sensitivity of steroid trajectories (Figure 4, bottom), the significance of noise within the PKA and G-protein cycles was reduced. In general, only interactions specific to SF1 activation and StAR transcription remained highly sensitive. For both scenarios, when sensitivity was averaged over all states or restricted to steroid production, steroidogenesis was only mildly sensitive due to uncertainty in T/E_2 _feedback and LH production (parameter numbers 84 - 93). Thus, uncertainty in the operator tuned parameters should have minimal effect on steroidogenic behavior.

### General behavior of the Steroidogenesis Model

**Figure 5:**
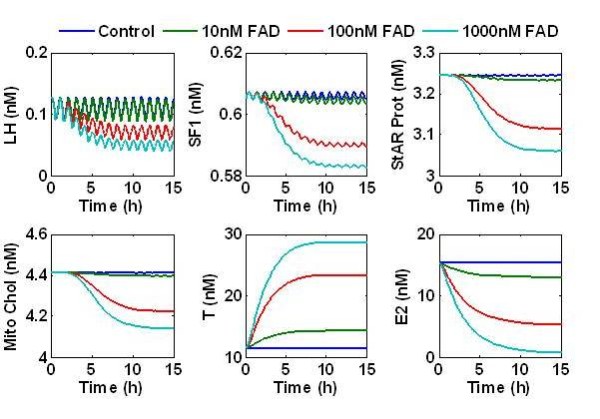
**The response of the steroidogenesis model to increasing FAD exposure. GnRH introduces a pulse affect on LH**. The oscillatory behavior at the LH receptor is filtered by the G-protein and PKA cycles and is significantly minimized by the time the signal reaches SF1. The cholesterol pool available inside the mitochondria is suppressed by FAD exposure. This leads to suppression of E_2 _and over production of T in the blood stream.

Several qualitative characteristics of LH signaling and steroid production were accurately captured by this mathematical description. Oscillatory dynamics imposed by the GnRH description was evident in the highly oscillatory behavior of proteins that were between the LH and SF1 activation (See Figure 5). The mean concentration and amplitude of the LH concentration was consistent with levels observed in mammals [38]. The magnitude of the oscillations was damped when the signal reached SF1 activation, and near fully damped at the level of StAR protein production. No significant oscillations were observable in the steroid concentrations. As FAD was introduced in the model, the E_2 _concentration in the blood stream was reduced. T in the blood stream was elevated during FAD exposure. The steady state concentration of T rises monotonically with FAD exposure level.

### Model Predictions of Steroid Dynamics

**Figure 6:**
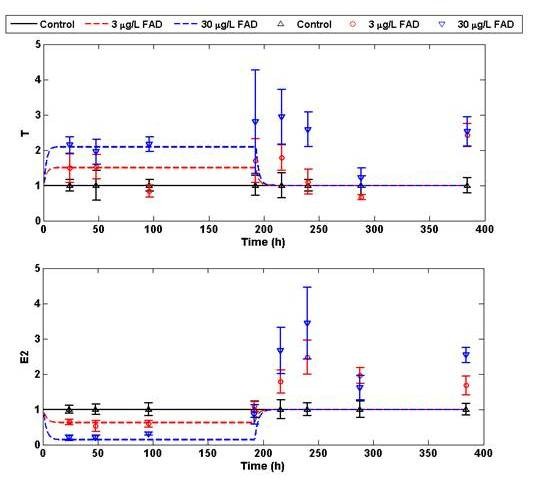
**Model predictions of FAD effects on E_2 _and T production over extended periods**. For 8 days, fish were exposed to 3 and 30 μg/L FAD. After the 8^th ^day FAD was flushed from the aquaria with fresh water. Fish continued to be sampled for 8 days to measure T and E_2 _during depuration. Data is shown in an upper triangle, open circle, and lower triangle for the control, 3, and 30 μg/L FAD exposures, respectively. The corresponding model predictions are solid black, dashed red, and dashed blue. Data from [39].

The model predictions of T and E_2 _were compared to published effects of FAD on E_2 _steroid production by ovaries in ex vivo assays [39]. Villeneuve et al [39] exposed FHM to 0, 3.0, or 30 μg FAD/L in aquaria and examined the impact of FAD on E_2 _and T production during 8 days of exposure and a subsequent 8 days of depuration (Figure 6). Behavior of mean levels of E_2 _were well predicted for all time points through 4 days of FAD exposure, but the model did not predict the apparent compensation occurring at day 8 of FAD exposure nor the subsequent over compensation of the steroidogenesis network after the stressor is removed. With the exception of day 4, the model was generally capable of predicting the mean levels of T production at 3 and 30 μg FAD/L during 8 d exposure, but, again, the model did not predict the overcompensation after stressor removal. At low levels of FAD (3 μg/L), T levels were not significantly different from controls due to sample variability.

### Microarray results

Global microarray expression analysis of in vivo and in vitro (ovary slices) tissues exposed to FAD revealed significantly different responses. Genes identified as differentially expressed (P < 0.05) within each treatment (FAD in vivo and in vitro) are listed in Additional File 1. In general, significantly more genes were affected in vivo than in vitro. When genes were filtered for those with 1.5-fold change or greater, in vitro FAD exposure resulted in 104 genes up regulated and 169 down regulated, whereas in vivo exposure resulted in 298 up regulated and 267 down regulated. Only 34 genes were common between the two exposures, suggesting that the steroidogenesis regulation involved in each system is very different when comparing ovary tissue to whole fish regulation.

To determine whether particular biological processes were enhanced or decreased in either exposure scenario, we calculated the over-representation of specific biological processes in the selected gene list. Ovaries exposed to FAD in vitro had 1 up- regulated and 24 down- regulated biological processes (including redundant categories) (P < 0.05), whereas in vivo exposures to FAD had 17 and 58 categories up- and down-regulated, respectively (P < 0.05) (Table 1).

**Table 1:** Functional analysis of genes differentially expressed in ovaries during 24 hr fadrozole exposure

ENRICHED GO TERMS UP-REGULATED FOR FAD IN VITRO		ENRICHED GO TERMS UP-REGULATED FOR FAD IN VIVO	
GO Name	p Value	GO Name	p Value
morphogenesis of an epithelium	0.011	pregnancy	0.009
protein amino acid glycosylation	0.029	ossification	0.012
glycoprotein biosynthesis	0.033	regulation of cell growth	0.012
biopolymer glycosylation	0.034	amino acid derivative catabolism	0.020
embryonic development (sensu Metazoa)	0.046	tissue development	0.021
glycoprotein metabolism	0.046	anti-apoptosis	0.027
		cell differentiation	0.029
		osteoblast differentiation	0.031
		development	0.036
		oogenesis	0.042
		reproductive physiological process	0.043
		nucleoside triphosphate metabolism	0.044

ENRICHED GO TERMS DOWN-REGULATED FOR FAD IN VITRO		ENRICHED GO TERMS DOWN-REGULATED FOR FAD IN VIVO	

GO Name	p Value	GO Name	p Value
gastrulation	0.008	negative regulation of apoptosis	0.002
DNA replication	0.009	anti-apoptosis	0.003
cellular carbohydrate metabolism	0.016	negative regulation of programmed cell death	0.003
carbohydrate metabolism	0.019	purine ribonucleotide biosynthesis	0.004
potassium ion transport	0.021	pregnancy	0.006
macromolecule biosynthesis	0.023	nucleoside triphosphate metabolism	0.007
glucose catabolism	0.025	transcription from RNA polymerase II promoter	0.009
hexose catabolism	0.026	regulation of physiological process	0.012
monosaccharide catabolism	0.026	signal transduction	0.013
positive regulation of cellular process	0.026	purine nucleoside triphosphate biosynthesis	0.015
alcohol catabolism	0.027	nucleoside triphosphate biosynthesis	0.016
physiological process	0.028	G-protein signaling, coupled to cyclic nucleotide second messenger	0.020
energy derivation by oxidation of organic compounds	0.032	reproductive organismal physiological process	0.021
mitochondrial transport	0.033	nucleoside metabolism	0.024
positive regulation of biological process	0.035	reproductive physiological process	0.024
glucose metabolism	0.036	energy coupled proton transport, down electrochemical gradient	0.024
protein amino acid glycosylation	0.040	ATP synthesis coupled proton transport	0.024
muscle contraction	0.042	regulation of cellular physiological process	0.026
protein biosynthesis	0.043	cell-cell adhesion	0.027
glycoprotein biosynthesis	0.044	cell proliferation	0.031
carbohydrate catabolism	0.044	regulation of programmed cell death	0.038
cellular carbohydrate catabolism	0.044	apoptosis	0.038
biopolymer glycosylation	0.045	G-protein signaling, coupled to cAMP nucleotide second messenger	0.038
cellular process	0.046	development	0.040

**The full list of significant GO terms are in Additional File 1.

In the FAD in vitro exposure (Table 1), epithelium morphogenesis, mitochondrial transport and protein amino acid glycosylation were among the most affected pathways. Biological processes involved in embryonic development, mitochondrial transport, carbohydrate-glucose metabolism, gastrulation and potassium transport were the most affected.

For the FAD in vivo exposure, among the most affected biological processes we found were skeletal-tissue remodeling-ossification pathways, purine nucleotide biosynthesis - deoxyribonucleotide metabolism, and oogenesis. Up-regulated biological processes were: skeletal- tissue remodeling - ossification (consistent with activation of the bone morphogenesis protein signaling pathways -and up regulation of Activin Receptor) and amino acid transport processes. Down were signal transduction - G-protein signaling, coupled to cAMP nucleotide second messenger, nucleotide -ATP biosynthesis, programmed cell death - anti-apoptosis, and pregnancy related processes.

### Predictions of observed genome regulation on steroid concentrations

**Figure 7:**
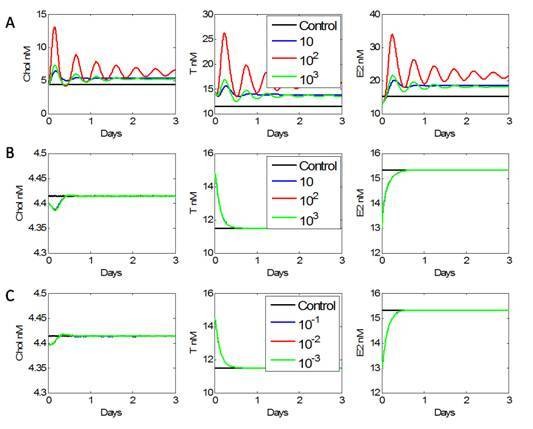
**Microarray data suggests that missing regulation involving (A) LH receptor up regulation, (B) Cytochrome P450c17αhydroxylase/17,20 lyase up regulation, or (C) inhibin down regulation may account for over production of T and E_2 _during depuration**. Each scenario was examined in the model by multiplying the appropriate parameter(s) with perturbations spanning three orders of magnitude. Neither up regulating P450 enzyme nor down regulating inhibin translation affected T or E_2 _production. Up regulating the translation of LH and/or increasing the available quantity of LH receptors both caused an increase in cholesterol translocation. This ultimately resulted in an over production of both E_2 _and T when compared to an unexposed fish (control).

Additional regulations observed in microarray data were simulated to predict the transcriptional regulation effects on protein behavior. It is assumed that up regulation of LH receptor results in a greater concentration of receptors, thus making the ovary cells more sensitive to the LH input, and resulting in an over-production of E_2 _and T in the exposed fish (Figure 7A). Similar results are obtained when the maximal rate of LH transcription was perturbed upwards. Perturbing concentrations of cytochrome P450-17αhydroxylase/17,20-lyase, identified as up-regulated in microarray data, had no observable affect on chol, T, or E_2 _dynamics (Figure 7B). The same is true of inhibin down-regulation (Figure 7C). All three possible transcriptional regulations were tested for possible synergistic effects, but none was observed.

## Discussion

The general approach to discerning stress response signatures in environmental toxicology is disease-specific and often based on the limited observations available. As more physiologically active compounds enter the environment, it becomes necessary to develop effective techniques to catalogue previous observations so as to extrapolate and predict the effects of new compounds. Mathematical models organize previous observations to facilitate hypothesis generation and validation. Here, a mathematical model was developed from literature and tested to predict steroid behavior during EDC exposure. The model then consolidated steroid dynamics observed during depuration with possible regulatory mechanisms identified via microarrays.

We developed the FHM female steroidogenesis model by merging G-proteins and PKA activation models with the architecture of a graphical steroidogenesis model [24,25,34,35]. The resulting model predicts several qualitative dynamics known to steroidogenesis (Figure 5). LH and cholesterol availability are the drivers for steroidogenesis. LH has pulsatile dynamics [35], which lead to oscillatory behavior in the G protein, PKA, and AC activation cycles in the model. The magnitude of the oscillations was suppressed as the steroidogenic signal traveled from the LH receptor to the SF1 activation site. They were further damped by the transcription and production of StAR, and no significant oscillations were observed in the steroid profiles. The oscillatory nature of LH signaling and the lack of oscillations in steroid concentrations have been measured in several studies, providing qualitative support to the models validity [24,35]. When inter-organ signaling was removed, the model reduced to the Breen *et al*. [24] in vitro model and was capable of reproducing in vitro steroid behavior observed during FAD exposure.

The model was further validated by its ability to predict E_2 _and T dynamics in response to different degrees of FAD stress. FAD is an ideal candidate for model discrimination because it specifically inhibits the aromatase activity responsible for converting E_1 _and T to E_2 _and therefore its effects in the steroidogenesis network can be suitably described. We monitored steroid production in fish exposed to 50 μg/L FAD for 6, 12, and 24 hrs and used this data for parameter training (Figure 2). The predictive capacity of the resulting model was validated against recently published data where FHM were monitored during exposures to 3 and 30 μg/L FAD for 8 days and a subsequent 8 day recovery period after the removal of FAD [39]. Testing showed that the model was capable of predicting steroid behavior to lower FAD exposures better than that of the training data (Figure 6). Model predictions and experimental results began to deviate at the 8d exposure period, when the steroidogenesis network appeared to start compensating for the FAD stress. This compensation was further revealed during depuration, when the network overcompensated after the stressor had been removed.

Sensitivity analysis supported PKA activation as the primary means of regulating the steroid response under stress. The parameter belonging to the PKA activation and AC activation could strongly control the steroid response (steroid concentrations are states #39, 45-55) by varying the cholesterol available to the inner mitochondria (Figure 3). Interestingly, enzyme mediated reactions responsible for the conversion of cholesterol to E_2 _and T were very robust to disturbances, making them unlikely candidates for missing signaling regulation elements. The sensitivity analysis for the model's nominal parameter set showed that steroidogenesis was robust (weakly sensitive) to perturbation in the LH pathway. Realizing that these results may be too strongly dependent on the nominal parameter selection, the sensitivity of each parameter was calculated for 1000 parameter generations, and analyzed for two different scenarios: (1) which parameters most affected the response of the entire steroidogenesis network (including signaling components) (2) which parameters most affected strictly the steroid trajectories (Figure 4). It is clear from the resulting sensitivity distributions that both the steroidogenesis network as a whole and specifically the steroid hormone profiles were most sensitive to disturbances or regulation effecting PKA inhibition and PKA translocation. Interestingly, the median of sensitivity of the steroid trajectories to PKA activation was suppressed when compared to the sensitivity of the entire network to PKA activation. Thus, sensitivity analysis implicated that PKA inhibition and translocation machinery were areas of interest when searching for missing regulation.

Contrary to the sensitivity analysis, microarray data suggested that mildly sensitive interactions were regulated to manipulate and control the steroid response during FAD stress. GO analysis showed several biological processes specific to reproduction and oogenesis were being modulated during stress, but the specific effects of this modulation on steroid behavior are difficult to determine. Focusing on transcriptional regulation observed within the ovary, LH receptor and P450 enzyme up-regulation and down-regulation of inhibin transcription in vivo are possible modes of stress compensation. By manipulating the corresponding parameters in the steroidogenesis model, each transcriptional regulation event was evaluated for its ability to explain over production of T and E_2 _during depuration (Figure 6). Increasing the total concentration of LH receptor resulted in over production of T and E_2 _during depuration, which was consistent with depuration data (Figure 7A). Similar dynamics were observed by manipulating the T regulation of LH mRNA transcription. Neither the up-regulation of CYP17 nor the down-regulation of inhibin had any effect on T and E_2 _production (Figure 7B and 7C).

Considering that both of these regulatory actions simultaneously produced no effect on steroid production, we hypothesize that the steroidogenesis network has evolved a distributed compensation strategy to FAD stress. Compensation is achieved by enhancing the sensitivity to extra-ovary signals from the pituitary/hypothalamus. This conclusion is further supported by in vitro microarray data that shows no significant intra-ovary regulation occurring (Additional File 1). This regulation is not readily observable during exposure since the FAD inhibition of aromatase activity dominates the network compensation efforts. During depuration, after the FAD stress has been removed, this regulation becomes apparent as fish begin to over produce T and E_2_.

These results challenge the traditional technique of employing sensitivity analysis to guide regulation discovery. Generally, elements within a network considered most sensitive are given priority during the next phase of experimentation and regulation exploration. Microarray data of the steroidogenesis network suggested that mildly sensitive parameters, rather than highly sensitive ones, were modulated during EDC stress response. This can be rationalized by further examining the noise and crosstalk that occurs in the G-protein, PKA, and AC cycles. Each of these cycles is involved in several biological processes. PKA is involved in the regulation of glyconeogenesis, glycolysis, and lipid metabolism [40] while the G-protein and AC has been associated with a vast number of essential biological processes [41]. In the absence of noise, these pathways can be manipulated to strongly control T and E_2 _production, but the ability to control steroid synthesis would be severely compromised by employing a highly sensitive target which is exposed to a great deal of cross-talk. The steroid network must balance sensitivity with robustness to noise. A moderately sensitive target which requires larger perturbations to account for compensation response may be less susceptible to intracellular noise. Thus, in silico exploration for potential regulation should consider targets that can effectively manipulate the behavior of interest while remaining robust to noise within the network.

## Conclusions

Elucidating regulatory machinery in complex biological systems can be aided by integrating heterogeneous data into consistent, mathematical frameworks so that causality between genomic variation and the ensuing steroid response can be examined. Furthermore, the implications of emergent properties, such as sensitivity and robustness, can expedite the discovery of regulatory mechanisms. Here, a signaling model of the FHM steroidogenesis network consolidates regulation observed in microarray data with its predicted effect on steroid production. The model ultimately identifies LH receptor regulation as a key compensation response to FAD stress. This result goes against the more accepted systems approach in which the most sensitive interactions are given priority in the search for undiscovered regulation. However, the traditional approach does not consider the significance of noise during regulation. Robust regulation is a careful balance of efficacy versus resistance to noise. The most sensitive interactions of the FHM steroidogenesis network are known to be highly communicative with other pathways, resulting in noisy control. LH receptor is a better means of compensation as hormone levels are highly regulated, guaranteeing that any compensation response will be controllable, though at the possible sacrifice of efficacy. In conclusion, the model of FHM steroidogenesis allows for the consolidation of genomic and physiological data, and ultimately illustrates that target prioritization must consider noise during discrimination.

## Methods

### Exposures

All fish used in the study were reproductively mature female FHM (5-6 months old) obtained from an on-site culture facility at the US EPA Mid-Continent Ecology Division (Duluth, MN). All laboratory procedures involving animals were reviewed and approved by the Animal Care and Use Committee in accordance with Animal Welfare Act and Interagency Research Animal Committee guidelines.

Exposures for in vivo experiments were conducted in 20 L glass aquaria containing 10 L of UV treated, membrane filtered, Lake Superior water containing nominal concentrations of 0 or 50 μg/L FAD. All treatments were delivered as a continuous flow through at a rate of approximately 45 ml/min without the use of carrier solvents. Toxicant (and control water) delivery was initiated to four replicate tanks per treatment group approximately 48 h prior to test initiation to ensure that stable water concentrations were achieved before adding fish. Exposures were then initiated by transferring random groups of 6 female FHM to each tank. After 6, 12 and 24 hr of exposure, two fish from each of 4 replicate tanks per treatment group were sampled (a total of 8 females per treatment per time point). During each sampling period, the fish were euthanized in a buffered solution of tricaine methanesulfonate (MS-222; Finquel; Argent, Redmond, WA, USA). Blood was collected using heparinized microhematocrit tubes and plasma was separated by centrifugation. A subsample of the ovary was used for an *ex vivo *steroid production assay (below). Plasma samples were stored at -80°C until extracted and analyzed. Gonads, brain, and pituitary were removed, snap frozen in liquid nitrogen, and stored at -80°C until needed for RNA extraction.

Ex vivo steroid production assays were conducted using methods adapted from McMaster et al. [42] as described previously [39]. Subsamples of ovary were transferred to glass test tubes containing 500 μl of Medium 199 (M2520; Sigma, St. Louis, MO, USA) supplemented with 0.1 mM isobutylmethylxanthine (IBMX; Sigma I7018) and 1 μg 25-hydroxycholesterol (Sigma)/ml, on ice. At the end of each sample collection period, the tubes were transferred to a 25°C shaker water bath and incubated for 12 h. Following incubation, the medium from each tube was collected and stored at -20°C until analyzed and the tissue subsample from each tube was removed and weighed. Tubes containing supplemented medium but no tissue were incubated, sampled, and analyzed along with experimental samples to serve as assay blanks.

In vitro exposures were conducted essentially as described for *ex vivo *assays. Briefly, 12 female FHM were euthanized as before and ovaries removed for in vitro testing. For each exposure, ovaries from 6 replicate fish were sliced into 12 ± 5 mg pieces and randomly distributed across sample culture wells to minimize sample effects due to potential tissue heterogeneity. Ovary slices were incubated in tissue culture medium supplemented with IBMX (0.1 mM), 25-hydroxycholesterol (1 μg/ml), and 50 uM FAD, or solvent control (0.07% methanol) for 12 hrs. Replicate ovary slices were removed, snap frozen in liquid nitrogen, and stored at -80°C until needed for RNA extraction. Medium from the incubations was used in analysis of E2 and T levels.

Steroids were extracted from medium samples (*ex vivo *and in vivo) or plasma samples by liquid-liquid extraction with diethyl ether and then quantified by radioimmunoassay [43].

### RNA extraction

RNA was isolated from tissue using Qiagen RNAeasy™ kits according to the manufacturer's instructions (Qiagen, Valencia, CA). RNA integrity and quantity were measured on an Agilent bioanalyzer (Agilent Technologies, Inc, Santa Clara, CA) and a nanodrop spectrophotometer (Nanodrop Technologies, Wilmington, DE), respectively.

### Microarray analysis

FHM 22,000 gene arrays (4 × 44 k format) were manufactured by Agilent and were purchased from EcoArray (Alachua, FL). Array hybridizations were performed using a reference design, where each sample was compared to a common reference sample. The reference consisted of equal amounts of RNA from female and male FHM tissues (liver, brain and gonad). Four replicates consisting of four different individuals were analyzed for each of the treatments (24 hr FAD in vivo and 12 hr FAD in vitro along with corresponding controls). cDNA synthesis, cRNA labeling and amplification and hybridization were performed following the manufacturer's kits and protocols (Agilent Low RNA Input Fluorescent Linear Amplification Kit and Agilent 60-mer oligo microarray processing protocol; Agilent, Palo Alto, CA). Briefly, a primer containing poly dT and a T7 polymerase promoter was added to 500 ng of total RNA. Reverse transcriptase was added to the reaction to synthesize the first and second strands of cDNA. Next, cRNA was synthesized from the double-stranded cDNA using T7 RNA polymerase, which simultaneously incorporates cyanine 3- (Cy3) or cyanine 5- (Cy5) labeled CTP (Perkin-Elmer, Boston, MA). The ovary samples were labeled with Cy5 while the reference sample was labeled with Cy3. Once the labeling was complete, samples were hybridized to the microarray for 17 hours. The microarrays were washed and scanned with a laser-based detection system (Agilent, Palo Alto, CA). MIAME compliant [44] text versions of microarray raw data have been deposited at the Gene Expression Omnibus website [GEO: GSE15924].

Microarray image processing and data pre-processing were performed using Agilent's Feature Extraction software v 9.5 (Agilent, 2007). The intensity of each spot was summarized by the median pixel intensity. A log2 transformed signal ratio between the experimental channel and the reference channel was calculated for each spot, followed by within array lowess transformation and between array scale normalization on median intensities. A two-way ANOVA was performed on log2 transformed signal ratios of each probe individually, followed by Tukey-HSD pair-wise comparisons to determine genes whose expression is significantly regulated by the treatments. Statistical significance was determined at a p-value of < = 0.05 with a FDR threshold of 16%. FDR was calculated using Benjamini-Hochberg approach [45]. Gene Ontology annotations were provided by EcoArray Inc. based on sequence homologies between FHM array probe target sequences and zebrafish, human and mouse genes. Over representation of differentially expressed genes in the biological process GO category was determined by Fisher Exact Test using a p-value < = 0.05 as a significance level cutoff.

### Steroidogenesis signaling model

The schematic of the steroidogenic signaling model is shown in Figure 1. In the ovary, cholesterol uptake from the outer mitochondrial membrane to the inner mitochondrial membrane is the rate limiting step. This transport process is primarily regulated via the StAR protein, which is in turn regulated by the LH pathway [46]. LH is secreted from the pituitary, binds the LH receptor in the theca cells of the ovary and activates the G-protein cycle. G-protein activation initiates a cascade of events, including adenylate cyclase (AC) activation, cyclic AMP (cAMP) activation, protein kinase A (PKA) activation, steroidogenic factor 1 (SF1) and StAR transcription. A LH signaling framework for activation of PKA has been reported by Bhalla and Hao *et al*. [34,47]. This framework was extended to describe the StAR protein activation via SF1. Cholesterol (Chol) uptake into the inner mitochondrial membrane is regulated by StAR protein and the concentration of cholesterol available to the outer mitochondrial membrane and is described by(1)

The first term of the right hand side is responsible for cholesterol uptake into the inner mitochondrial membrane and second term is the subsequent consumption of inner mitochondrial cholesterol by steroid synthesis.

The expression of SF1 and activated SF1 was considered comparable to cAMP's activation of CREB protein, as reported by Hao et al. [34]. Also, similar kinetic descriptions reported for *per *gene transcription and translation were applied to StAR mRNA and StAR protein synthesis.

The LH signaling architecture was coupled with the steroidogenesis enzymatic network established by Breen *et al*. [24], where cholesterol is utilized for synthesis of subsequent steroid products. It is assumed that T, AD, estrone E_1_, and E_2 _are secreted into the blood stream/plasma with the following kinetic descriptions:(2)(3)(4)(5)

A number of EDCs can inhibit steroidogenic enzymes. The predictive power of the model is tested by stressing ovary cells with FAD. FAD is a specific competitive inhibitor of aromatase (CYP19), which catalyzes two important steroidogenic metabolic reactions: AD to E_1 _and T to E_2 _[24,48]. FAD diffuses into the ovary and maintains equilibrium with medium as:(6)

*FAD*_*ex *_is FAD concentration in the bloodstream/plasma and *FAD *is the concentration in the ovary. Parameter *km*_*15 *_is the partition coefficient constant and *km*_*20 *_is the first order diffusion constant from the ovary to the medium. The FAD inhibition is incorporated by modulating the kinetic parameters responsible for the conversion of AD to E_1 _and T to E_2_. (See Additional files 2, 3, 4 for a list of all ODEs, parameters and parameter information).

E_2 _and T are circulated to other steroidogenic organs by the blood stream. LH is secreted from pituitary and controlled by Gonadotropin-releasing hormone. LH release is further regulated, via negative feedback, by T and E_2 _[49-54]. This regulation is lumped into a module labeled signaling modulation since a more detailed model of the pituitary and hypothalamus are not available at this time. GnRH, E_2 _and T feedback is described as(7)

where *L*_*m *_is the amount of LH mRNA. GnRH is modeled as a pulsatile forcing function with a magnitude of 10 nM and a 30 minute pulse occurring one time per hour.

### Parameter selection

Parameters *k*_*1 *_through *k*_*60 *_and parameters for StAR mRNA, StAR protein, LH mRNA and LH protein were obtained from Hao et al [34]. Half of the parameters specific to the enzyme-mediated conversion of cholesterol to its derivatives (*km*_*9*_, *km*_*10*_, ... *km*_*20*_) are from an in vitro study of ovarian steroidogenesis [24]. During data analysis, concentrations of the sex steroids within 1 - 2 nM are observed. A flux balance analysis (FBA) was performed to predict the unmeasured, intermediate steroids concentrations. The analysis was performed such as to maximize T and E_2 _production (See Additional File 5). The remaining parameters were tuned to achieve the steroid response observed when fish were exposed to 50 μg/L FAD for 24 hours (See Figure 2), and to match the intermediate steroid profiles predicted by the FBA. Care was taken to ensure the parameter values were on the order of those observed in the Breen *et al*. [24] model. Initial conditions for an unexposed FHM were established by determining the steady state of the system applying the initial conditions from [34] with the additional constraint of a concentration of 60,000 nM cholesterol outside of the mitochondria. The resulting steady state values are in Additional File 6. Differential equations were solved using the MATLAB stiff ODE integrator ode15s. It should be noted that stiffness was not an issue while simulating the nominal model.

### Sensitivity analysis

Sensitivity is the response of a state trajectory to infinitesimal perturbations in parameter values, and this measure is normalized by the size of the state and parameter so that comparisons between states can be made. For a lumped system model described by , the relative sensitivity is defined as:(8)

where *x *is the state vector and *p *is the parameter vector. Sensitivity is calculated using finite differences.

Sensitivity is a local measure whose results may be highly dependent on the choice of nominal parameters. To approximate a semi-global understanding of the effects of parameter variability on the steroid response, the relative sensitivity was calculated for each state to each parameter included in the steroidogenesis model for 1000 parameter generations. Each parameter generation was created by sampling a relative perturbation value from a normal distribution centered about zero with a standard deviation of 10.0%. For each generation, 114 perturbations were sampled from the distribution and applied in a multiplicative fashion to each of the 114 parameters in the model (i.e., parameter *k*_*i *_for generation *j *is *k*_*i,j *_= *k*_i_*(1 + *δ*_*j*_) where *δ*_*j *_is the perturbation). The initial conditions for each calculation are the same as those applied for the nominal model. Each sensitivity trajectory was calculated over a span of 30 days. Figure 3 shows the sensitivity of each state to each parameter, averaged over time, for a *single *generation.

A boxplot was used to demonstrate the robustness of the results of Figure 3. Boxplots illustrate statistical differences between competing hypotheses. Referring to Figure 4, the lower, middle-lower, middle-upper, and upper boxes represent the lower quartile, mid-lower quartile, mid-upper quartile, and upper quartile of a distribution. For each parameter, the sensitivity of that parameter is averaged over time and states for each generation, resulting in 1000 average relative sensitivity (ARS) values for each parameter. The boxplots illustrate how the ARS is distributed for each parameter. The red bar represents the median ARS for each parameter. If the notches about the median of two parameters do not overlap, one can conclude with 95% confidence that the true medians differ. Figure 4 shows two scenarios. The top is when the sensitivity of all states is considered during averaging. The bottom is when only chol and steroid concentrations are considered. It is known that, in biological systems, parameter values may fluctuate over orders of magnitude, but discretization issues and stiffness arising in particular parameter generations made it difficult to consider larger uncertainty ranges in a timely manner. The distributions of the ARS showed that parameter rankings (most sensitive versus least sensitive) were well maintained despite changes to parameter value, and previous studies have shown similar conclusions [14,15,55]

### Simulation studies

Expression variation observed in microarray data was simulated to predict the effect of transcriptional regulation on protein behavior. It is not safe to assume that up or down genomic regulation translates linearly with the corresponding protein concentration. The affect of each gene on steroidogenic behavior is evaluated by perturbing the corresponding enzyme concentration (or activity) in the direction of the reported regulation over several orders of magnitude. We used as a control the simulation results of an unexposed fish. The initial concentration of LH receptor was multiplied by dimensionless perturbations of [10^1^, 10^2^, 10^3^] to simulate LH receptor up regulation (Figure 7A). Cytochrome P450c17αhydroxylase/17,20 lyase, identified as up regulated in microarray data, impacted the conversion rates, k_5 _and k_7_, respectively. Both were multiplied by order of magnitude perturbations, [10^1^, 10^2^, 10^3^] dimensionless. Inhibin down regulation was achieved by multiplying the maximum inhibin production rate (V_spin_) by order of magnitude perturbations [10^-1^, 10^-2^, 10^-3^] dimensionless units (Figure 7C).

## Abbreviations

AC: Adenylate cyclase; AD: Androstenedione; ARS: Average relative sensitivity; Chol: Cholesterol; DHEA: Dehydroepiandrosterone; EDC: Endocrine disrupting compound; FAD: Fadrozole; FHM: Fathead Minnow; E1: Estrone; E2: Estradiol; HPREG: 17 αhydroxypregnenolone; HPROG: 17 α hydroxyprogesterone; LH: Luteinizing hormone; PKA: Protein kinase A; PREG: Pregnenolone; PROG: Progesterone; T: Testosterone.

## Authors' contributions

JES carried out the model analysis/development and testing, and drafted the manuscript. KG was primary model developer. NGR carried out the microarray experiments and helped to draft the manuscript. LL performed the microarray statistical analysis. DLV performed the fish exposures and ex vivo and in vitro studies. Both EJP and FJD conceived of the study, and participated in its design and coordination and helped to draft the manuscript. All authors read and approved the final manuscript

## Supplementary Material

Additional file 1 **Functional Gene Ontology analysis of genes differentially expressed in ovaries during fadrozole exposure**. A list of all significantly enriched Gene Ontology (GO) categories from in vitro exposures of ovary slices and in vivo exposures of ovaries to fadrozole. This list contains the number of genes selected, the number of genes on the array, the Fisher p Value, and the false discovery rate for each GO category. Click here for file

Additional file 2 **Chemical Species of the Steroidogenesis Model**. A list and full definition of all chemical species used in the modeling annotation of the Steroidogenesis Model. Click here for file

Additional file 3 **Rate equations for ovarian steroidogenesis**. A complete listing of all 55 rate equations used in development of the steroidogenesis model. Click here for file

Additional file 4 **Steroidogenesis Model Parameters**. A complete listing of parameters, parameter descriptions, parameter units and values used in development of the Steroidogenesis model. Click here for file

Additional file 5 **Flux balance analysis of ovarian steroidogenesis model**. This file describes how Flux Balance Analysis was conducted. Click here for file

Additional file 6 **Initial model conditions**. This file describes the initial condition values for each variable within the model. Click here for file
