# Supplementary material for: Fathead minnow steroidogenesis: in silico analyses reveals tradeoffs between nominal target efficacy and robustness to cross-talk
Source: BMC Syst Biol. 2010 Jun 28;4:89. doi: 10.1186/1752-0509-4-89 (PMC2905341; doi:10.1186/1752-0509-4-89)
Supplement: Additional file 1 — Functional Gene Ontology analysis of genes differentially expressed in ovaries during fadrozole exposure. A list of all significantly enriched Gene Ontology (GO) categories from in vitro exposures of ovary slices and in vivo exposures of ovaries to fadrozole. This list contains the number of genes selected, the number of genes on the array, the Fisher p Value, and the false discovery rate for each GO category. [file 1752-0509-4-89-S1.DOC]

# Additional File 1 - Functional analysis of genes differentially expressed in ovaries during 24 hr fadrozole exposure.
