# Supplementary material for: Fathead minnow steroidogenesis: in silico analyses reveals tradeoffs between nominal target efficacy and robustness to cross-talk
Source: BMC Syst Biol. 2010 Jun 28;4:89. doi: 10.1186/1752-0509-4-89 (PMC2905341; doi:10.1186/1752-0509-4-89)
Supplement: Additional file 2 — Chemical Species of the Steroidogenesis Model. A list and full definition of all chemical species used in the modeling annotation of the Steroidogenesis Model. [file 1752-0509-4-89-S2.DOC]

# Additional File 2 - Chemical Species of the Steroidogenesis Model

| *AC:* | Adenylate cyclase. |
| --- | --- |
| *Gas.AC:* | Gs activated adenylate cyclase. |
| *GTP.Gas:* | Activated Gsalpha. |
| *cAMP:* | Cyclic AMP. |
| *Gas.AC.ATP:* | ATP bound Gs activated adenylate cyclase. |
| *R2C2:* | Protein Kinase A heterotetramer with two molecules of regulatory subunits and two molecules of catalytic subunits. |
| *cAMP.R2C2:* | One molecule of cAMP bound PKA heterotetramer. |
| *cAMP2.R2C2:* | Two molecules of cAMP bound PKA heterotetramer. |
| *cAMP3.R2C2:* | Three molecules of cAMP bound PKA heterotetramer. |
| *cAMP4.R2C2:* | Four molecules of cAMP bound PKA heterotetramer. |
| *cAMP-PDE:* | cAMP phosphodiesterase. |
| *cAMP-PDE_PDE-c:* | cAMP bound cAMP phosphodiesterase complex. |
| *cAMP-PDE*:* | Phosphorylated cAMP phosphodiesterase. |
| *cAMP-PDE*_PDE*_c:* | cAMP bound phosphorylated cAMP phosphodiesterase complex. |
| *PKA-active_p_PDE_c:* | Activated PKA and cAMP phosphodiesterase complex*.* |
| *PKA-active_complex:* | Cytoplasmic activated PKA. |
| *cAMP4.R2C:* | Four molecules of cAMP bound PKA with one catalytic subunit released. |
| *cAMP4.R2:* | Four molecules of cAMP bound PKA with both catalytic subunits released. |
| *Gbg:* | G-protein beta and gamma subunits. |
| *L.R.GDP.Gasbg:* | Ligand bound receptor-G-protein complex. |
| *GDP.Gas:* | GDP bound Gsalpha. |
| *GDP.Gasbg:* | GDP bound Gs trimeric complex. |
| *R.GDP.Gasbg:* | Receptor-G-protein complex. |
| *Inhibited-PKA_c:* | Inhibitor bound cytoplasmic PKA. |
| *PKA-inhibitor_c:* | Cytoplasmic PKA inhibitor. |
| *R2:* | PKA regulatory unit dimer. |
| *R2C:* | PKA heterotetramer reforming intermediate. |
| *Inhibited-PKA_n:* | Inhibitor bound nuclear PKA. |
| *L.R:* | LH Ligand bound receptor. |
| *R:* | Receptor. |
| *PKA-active_n:* | Nuclear active PKA. |
| *PKA-inhibitor_n:* | Nuclear PKA inhibitor. |
| *SF1:* | Inactive form transcription factor SF1. |
| *SF1.PKA-active_n:* | SF1 and nuclear active PKA complex. |

| *SF1*:* | Phosphorylated active form SF1. |
| --- | --- |
| *SF1*0:* | Basal phosphorylated active form SF1. |
| *StARm:* | *StAR mRNA.* |
| *StARp* | *StAR* protein |
| *Chol_O_* | Cholesterol outer mitochondrial membrane |
| *Chol_i_* | Cholesterol inner mitochondrial membrane |
| *L_m_* | Luteinizing hormone (LH) mRNA |
| *L* | Luteinizing hormone (LH) protein |
| *GnRH* | Gonadotropin releasing hormone |
| *T* | Testosterone |
| *PERG* | Pregnenolone |
| *HPREG* | 17-Hydroxypregnenolone |
| *PROG* | Progesterone |
| *HPROG* | 17-Hydroxyprogesterone |
| *AD* | Androstenedione |
| *E1* | Estrone |
| *E2* | Estradiol |
| *DHEA* | Dehydroepiandrosterone |
| *T_ex_* | Plasma T |
| *AD_ex_* | Plasma AD |
| *E1_ex_* | Plasma E1 |
| *E2_ex_* | Plasma E2 |
| *FAD* | Fadrazole in plasma |
| *FADex* | Fadrazole in media |
