# Supplementary material for: Fathead minnow steroidogenesis: in silico analyses reveals tradeoffs between nominal target efficacy and robustness to cross-talk
Source: BMC Syst Biol. 2010 Jun 28;4:89. doi: 10.1186/1752-0509-4-89 (PMC2905341; doi:10.1186/1752-0509-4-89)
Supplement: Additional file 3 — Rate equations for ovarian steroidogenesis. A complete listing of all 55 rate equations used in development of the steroidogenesis model. [file 1752-0509-4-89-S3.DOC]

**Additional file 3 - Rate equations for ovarian steroidogenesis**

1. Equations 1 – 31 are from [34]. SF1 is considered activated instead of CREB.
2. Equations 40 -55 are from [24].

$$\frac{d[AC]}{dt}=k_{16}[Gas.AC]+k_{43}[Gas.AC]-k_{15}[AC][GTP.Gas]$$

$$\frac{d[cAMP]}{dt}=k_{12}[Gas.AC\_cyclase\_complex]-k_{17}[cAMP][R2C2]+k_{18}[cAMP.R2C2]$$

$$-k_{19}[cAMP][cAMP.R2C2]+k_{20}[cAMP2.R2C2]-k_{21}[cAMP][cAMP2.R2C2]+k_{22}[cAMP3.R2C2]-$$

$$k_{23}[cAMP][cAMP3.R2C2]+k_{24}[cAMP4.R2C2]-k_{35}[cAMP][cAMP-PDE]$$

$$\begin{matrix} +k_{36}[cAMP-PDE\_PDE\_complex]- \\ k_{38}[cAMP][cAMP-PDE*]+k_{39}[cAMP-PDE*\_PDE*\_complex] \end{matrix}$$

$$\frac{d[cAMP-PDE]}{dt}=k_{31}[PKA-active\_phosph-PDE\_complex]-k_{33}[cAMP-PDE][PKA-active\_c]$$

$$+k_{34}[cAMP-PDE*]-k_{35}[cAMP][cAMP-PDE]+k_{36}[cAMP-PDE\_PDE\_complex]$$

$$+k_{37}[cAMP-PDE\_PDE\_complex]$$

$$\frac{d[cAMP-PDE*]}{dt}=k_{32}[PKA-active\_phosph-PDE\_complex]-k_{33}[cAMP-PDE][PKA-active\_c]$$

$$-k_{34}[cAMP-PDE*]-k_{38}[cAMP][cAMP-PDE*]+k_{39}[cAMP-PDE*\_PDE*\_complex]+$$

$$k_{40}[cAMP-PDE*\_PDE*\_complex]$$

$$\frac{d[cAMP.R2C2]}{dt}=k_{17}[cAMP][R2C2]-k_{18}[cAMP.R2C2]+k_{19}[cAMP][cAMP.R2C2]+k_{20}[cAMP2.R2C2]$$

$$\frac{d[cAMP2.R2C2]}{dt}=k_{19}[cAMP][cAMP.R2C2]-k_{20}[cAMP2.R2C2]-k_{21}[cAMP][cAMP2.R2C2]$$

$$+k_{22}[cAMP3.R2C2]$$

$$\frac{d[cAMP3.R2C2]}{dt}=k_{21}[cAMP][cAMP2.R2C2]-k_{22}[cAMP3.R2C2]-k_{23}[cAMP][cAMP3.R2C2]$$

$$+k_{24}[cAMP4.R2C2]$$

$$\frac{d[cAMP4.R2]}{dt}=k_{28}[cAMP4.R2C]-k_{27}[cAMP4.R2][PKA-active\_c]-k_{60}[cAMP4.R2]$$

$$\frac{d[cAMP4.R2C]}{dt}=k_{26}[cAMP4.R2C2]-k_{25}[cAMP4.R2C][PKA-active\_c]-k_{28}[cAMP4.R2C]+$$

$$k_{27}[cAMP4.R2][PKA-active\_c]$$

$$\frac{d[cAMP4.R2C2]}{dt}=k_{23}[cAMP][cAMP3.R2C2]-k_{24}[cAMP4.R2C2]-k_{26}[cAMP4.R2C2]+$$

$$k_{25}[cAMP4.R2C][PKA-active\_c]$$

$$\frac{d[GDP.Gas]}{dt}=k_{11}[GTP.Gas]-k_{9}[Gbg][GDP.Gas]+k_{43}[Gas.AC]$$

$$\frac{d[Gbg]}{dt}=k_{10}[L.R.GDP.Gasbg]-k_{9}[Gbg][GDP.Gas]+k_{41}[GDP.Gasbg]$$

$$\frac{d[GDP.Gasbg]}{dt}=k_{9}[Gbg][GDP.Gas]-k_{3}[GDP.Gasbg][L.R]+k_{4}[L.R.GDP.Gasbg]-k_{5}[GDP.Gasbg][R]$$

$$+k_{6}[R.GDP.Gasbg]-k_{41}[GDP.Gasbg]$$

$$\frac{d[Gas.AC]}{dt}=k_{15}[AC][GTP.Gas]-k_{16}[Gas.AC]-k_{13}[Gas.AC][ATP]+k_{12}[Gas.AC\_cyclase\_complex]$$

$$+k_{14}[Gas.AC\_cyclase\_complex]-k_{43}[Gas.AC]$$

$$\begin{matrix} \frac{d[GTP.Gas]}{dt}=k_{10}[L.R.GDP.Gasbg]-k_{11}[GTP.Gas]- \\ k_{15}[AC][GTP.Gas]+k_{16}[Gas.AC]+k_{41}[GDP.Gasbg] \end{matrix}$$

$$\frac{d[inhibited-PKA\_c]}{dt}=k_{29}[PKA-active\_c][PKA-inhibitor\_c]-k_{30}[inhibited-PKA\_c]-$$

$$k_{53}[inhibited-PKA\_c][R2]+k_{54}[PKA-inhibitor\_c][R2C]-k_{55}[inhibited-PKA\_c][R2C]$$

$$+k_{56}[PKA-inhibitor\_c][R2C2]+k_{51}[inhibited-PKA\_n]-k_{52}[inhibited-PKA\_c]$$

$$\frac{d[L.R]}{dt}=k_{1}[L][R]-k_{2}[L.R]-k_{3}[GDP.Gasbg][L.R]+k_{4}[L.R.GDP.Gasbg]+k_{10}[L.R.GDP.Gasbg]$$

$$-k_{42}[L.R]$$

$$\frac{d[R]}{dt}=-k_{1}[L][R]+k_{2}[L.R]-k_{5}[R][GDP.Gasbg]+k_{6}[R.GDP.Gasbg]$$

$$\frac{d[R.GDP.Gasbg]}{dt}=k_{5}[R][GDP.Gasbg]-k_{6}[R.GDP.Gasbg]-k_{7}[L][R.GDP.Gasbg]+k_{8}[L.R.GDP.Gasbg]$$

$$\frac{d[L.R.GDP.Gasbg]}{dt}=k_{3}[GDP.Gasbg][L.R]-k_{4}[L.R.GDP.Gasbg]-k_{10}[L.R.GDP.Gasbg]$$

$$+k_{7}[L][R.GDP.Gasbg]-k_{8}[L.R.GDP.Gasbg]$$

$$\frac{d[PKA-active\_c]}{dt}=k_{26}[cAMP4.R2C2]-k_{25}[cAMP4.R2C][PKA-active\_c]+k_{28}[cAMP4.R2C]-$$

$$k_{27}[cAMP4.R2][PKA-active\_c]+k_{31}[PKA-active\_phosh-PDE\_complex]-$$

$$k_{33}[cAMP-PDE][PKA-active\_c]-k_{29}[PKA-active\_c][PKA-inhibitor\_c]+$$

$$k_{30}[inhibited-PKA\_c]+k_{32}[PKA-active\_phosph-PDE\_complex-k_{44}[PKA\_active\_c]$$

$$+k_{45}[PKA-active\_n]$$

$$\frac{d[PKA-inhibitor\_c]}{dt}=-k_{29}[PKA-active\_c][PKA-inhibitor\_c]+k_{30}[inhibited-PKA\_c]$$

$$-k_{57}[PKA-inhibitor\_c]+k_{58}[PKA-inhibitor\_n]+k_{55}[inhibited-PKA\_c][R2C]-$$

$$k_{56}[PKA-inhibitor\_c][R2C2]+k_{53}[inhibited-PKA\_c][R2]-k_{54}[PKA-inhibitor\_c][R2C]$$

$$\frac{d[R2C2]}{dt}=k_{55}[inhibited-PKA\_c][R2C]-k_{56}[PKA-inhibitor\_c][R2C2]-k_{17}[cAMP][R2C2]$$

$$+k_{18}[cAMP.R2C2]$$

$$\frac{d[Gas.AC\_cyclase\_complex]}{dt}=k_{13}[Gas.AC][ATP]-k_{12}[Gas.AC\_cyclase\_complex]$$

$$-k_{14}[Gas.AC\_cyclase\_complex]$$

$$\frac{d[cAMP-PDE\_PDE\_complex]}{dt}=k_{35}[cAMP][cAMP-PDE]-k_{36}[cAMP-PDE\_PDE\_complex]$$

$$-k_{37}[cAMP-PDE\_PDE\_complex]$$

$$\begin{matrix} \frac{d[cAMP-PDE*\_PDE*\_complex]}{dt}=k_{38}[cAMP][cAMP-PDE*]- \\ k_{39}[cAMP-PDE*\_PDE*\_complex]- \end{matrix}$$

$$k_{40}[cAMP-PDE*\_PDE*\_complex]$$

$$\frac{d[PKA-active\_phosph-PDE\_complex]}{dt}=-k_{31}[PKA-active\_phosph-PDE\_complex]$$

$$+k_{33}[cAMP-PDE][PKA-active\_c]-k_{32}[PKA-active\_phosph-PDE\_complex]$$

$$\begin{matrix} \frac{d[inhibited-PKA\_n]}{dt}=-k_{51}[inhibited-PKA\_n]+k_{52}[inhibited-PKA\_c]- \\ k_{50}[inhibited-PKA\_n] \end{matrix}$$

$$+k_{49}[PKA-active\_n][PKA-inhibitor\_n]$$

$$\frac{d[PKA-inhibitor\_n]}{dt}=k_{50}[inhibited-PKA\_n]-k_{49}[PKA-active\_n][PKA-inhibitor\_n]+$$

$$k_{57}[PKA-inhibitor\_c]-k_{58}[PKA-inhibitor\_n]$$

$$\frac{d[R2]}{dt}=k_{60}[cAMP4.R2]-k_{53}[inhibited-PKA\_c][R2]+k_{54}[PKA-inhibitor\_c][R2C]$$

$$\begin{matrix} \frac{d[R2C]}{dt}=k_{53}[inhibited-PKA\_c][R2]-k_{54}[PKA-inhibitor\_c][R2C]- \\ k_{55}[inhibited-PKA\_c][R2C] \end{matrix}$$

$$+k_{56}[PKA-inhibitor\_c][R2C2]$$

$$\begin{matrix} \frac{d\left[PKA-active\_n\right]}{dt}=k_{44}\left[PKA-active\_c\right]-k_{45}\left[PKA-active\_n\right]- \\ k_{46}[SF1][PKA-active\_n]+ \end{matrix}$$

$$k_{47}\left[SF1.PKA-active\_n\right]+k_{48}\left[SF1.PKA-active\_n\right]-k_{49}\left[PKA-active\_n\right]\left[PKA-inhibitor\_n\right]$$

$$+k_{50}\left[inhibited-PKA\_n\right]$$

$$\frac{d\left[SF1\right]}{dt}=k_{47}\left[SF1.PKA-active\_n\right]-k_{46}\left[SF1\right]\left[PKA-active\_n\right]+k_{59}\left[SF1\right]$$

$$\frac{d\left[SF1*\right]}{dt}=k_{48}\left[SF1.PKA-active\_n\right]-k_{59}\left[SF1*\right]$$

$$\begin{matrix} \frac{d\left[SF1.PKA-active\_n\right]}{dt}=k_{46}\left[SF1\right]\left[PKA-active\_n\right]-k_{47}\left[SF1.PKA-active\_n\right] \\ -k_{48}\left[SF1.PKA-active\_n\right] \end{matrix}$$

$$\frac{d[StARm]}{dt}=V_{spSTRA}\frac{[SF1*]^{n}}{K_{spSTAR}^{n}+[SF1*]^{n}}-V_{mpSTAR}\frac{[StARm]}{K_{mpSTAR}+[StARm]}-K_{dmpSTAR}[StARm]$$

$$\frac{d\left[StAR_{p}\right]}{dt}=V_{prSTAR}\frac{\left[StARm\right]}{K_{prSTAR}+\left[StARm\right]}-K_{dprSTAR}\left[StAR_{p}\right]$$

$$\frac{d[L_{m}]}{dt}=V_{spLH}\frac{[GnRH]^{n}}{KcpLH+[GnRH]^{n}+\frac{[T_{ex}]^{2}}{K_{inLH}}+\frac{[E2_{ex}]^{2}}{K_{inLH}}}-V_{mpLH}\frac{[L_{m}]}{K_{mpLH}+[L_{m}]}-K_{dmpLH}[L_{m}]$$

$$\frac{d\left[L\right]}{dt}=V_{prLH}\frac{\left[L_{m}\right]}{K_{prLH}+\left[L_{m}\right]}-K_{dprLH}\left[L\right]$$

$$\frac{d[Chol_{o}]}{dt}=0$$

$$\frac{d[Chol_{i}]}{dt}=V_{ch}\frac{[StAR_{p}]}{StAR_{p}^{\max}-StAR_{p}}\frac{[Chol_{o}]}{K_{ch}+[Chol_{0}]}-km1[Chol_{i}]$$

$$\frac{d[PREG]}{dt}=km1 [Chol_{i}]-(km2+km4) [PREG]$$

$$\frac{d[HPREG]}{dt}=km2 [PREG]-(km3+km5) [HPREG]$$

$$\frac{d[DHEA]}{dt}=km3 [HPREG]-km6 [DHEA]$$

$$\frac{d[PROG]}{dt}=km4 [PREG]-km7 [PROG]$$

$$\frac{d[HPROG]}{dt}=km5 [HPREG]+km7 [PROG]-km8 [HPROG]$$

$$\frac{d[AD]}{dt}=km5 [DHEA]+km8 [HPROG]-(km9+km18+\frac{km11}{\alpha_{1}}) [AD]$$

$$\frac{d[T]}{dt}=km9 [AD]-(km10+\frac{km12}{\alpha_{2}}) [T]$$

$$\frac{d[E1]}{dt}= \frac{km11}{\alpha_{1}} [AD]-(km13+km19)[E1]$$

$$\frac{d[E2]}{dt}=km13 [E1]+\frac{km12}{\alpha_{2}}[T]-km14 [E2]$$

$$\frac{d[AD_{ex}]}{dt}=km18 [AD]$$

$$\frac{d[E1_{ex}]}{dt}=km19 [E1]$$

$$\frac{d[T_{ex}]}{dt}=km10 [T]$$

$$\frac{d[E2_{ex}]}{dt}=km14 [E2]$$

$$\frac{d[FAD]}{dt}=km15 [FAD_{ex}]-k20[FAD]$$
