# Supplementary material for: Fathead minnow steroidogenesis: in silico analyses reveals tradeoffs between nominal target efficacy and robustness to cross-talk
Source: BMC Syst Biol. 2010 Jun 28;4:89. doi: 10.1186/1752-0509-4-89 (PMC2905341; doi:10.1186/1752-0509-4-89)
Supplement: Additional file 4 — Steroidogenesis Model Parameters. A complete listing of parameters, parameter descriptions, parameter units and values used in development of the Steroidogenesis model. [file 1752-0509-4-89-S4.DOC]

**Additional file 4 - Steroidogenesis Model Parameters**

Parameters k1 through KinLH below are from [34].

| Parameter | Units | Value | Description |
| --- | --- | --- | --- |
| *k1* | nM-1.s-1 | 0.0001 | kf for ligand binding to standalone receptors. |
| *k2* | s-1 | 0.1 | kb for ligand binding to standalone receptors. |
| *k3* | nM-1.s-1 | 0.001 | kf for G-protein coupling to ligand-receptor complex. |
| *k4* | s-1 | 0.3 | kb for G-protein coupling to ligand-receptor complex |
| *k5* | nM-1.s-1 | 0.0002 | kf for G-protein coupling to receptor. |
| *k6* | s-1 | 0.1 | kb for G-protein coupling to receptor. |
| *k7* | nM-1.s-1 | 0.01 | kf for Ligand binding to receptor G-protein complex |
| *k8* | s-1 | 0.1 | kb for ligand binding to receptor G-protein complex |
| *k9* | nM-1.s-1 | 0.006 | kf for G-protein subunit association. |
| *k10* | s-1 | 0.025 | kf for G-protein activation |
| *k11* | s-1 | 0.066667 | kf for intrinsic GTP hydrolysis. |
| *k12* | s-1 | 0.00214275 | k3 for cAMP production by activated Adenylate cyclase. |
| *k13* | nM-1.s-1 | 0.0045 | k1 for ATP binding to activated AC. |
| *k14* | s-1 | 90 | k2 for ATP and AC dissociation. |
| *k15* | nM-1.s-1 | 0.25 | kf for activated G protein binding to AC. |
| *k16* | s-1 | 1 | kb for activated G-protein binding to AC. |
| *k17* | nM-1.s-1 | 0.027 | kf for cAMP binding to PKA regulatory site B1. |
| *k18* | s-1 | 33 | kb for cAMP binding to PKA regulatory site B1. |
| *k19* | nM-1.s-1 | 0.108 | kf for cAMP binding to PKA regulatory site B2. |
| *k20* | s-1 | 0.66 | kb for cAMP binding to PKA regulatory site B2. |
| *k21* | nM-1.s-1 | 0.108 | kf for cAMP binding to PKA regulatory site A1. |
| *k22* | s-1 | 0.66 | kb for cAMP binding to PKA regulatory site A1. |
| *k23* | nM-1.s-1 | 0.216 | kf for cAMP binding to PKA regulatory site A2. |
| *k24* | s-1 | 0.325 | kb for cAMP binding to PKA regulatory site A2. |
| *k25* | nM-1.s-1 | 0.108 | kb for releasing PKA catalytic unit C1. |
| *k26* | s-1 | 1.40625 | kf for releasing PKA catalytic unit C1. |
| *k27* | nM-1.s-1 | 0.108 | kb for releasing PKA catalytic unit C2. |
| *k28* | s-1 | 1.40625 | kf for releasing PKA catalytic unit C2. |
| *k29* | nM-1.s-1 | 0.060 | kf for cytoplasmic inhibitor binding to active PKA. |
| *k30* | s-1 | 0.1 | kb for cytoplasmic inhibitor binding to active PKA. |
| *k31* | s-1 | 3.6 | k2 for PKA phosphorylating PDE. |
| *k32* | s-1 | 0.9 | k3 for PKA phosphorylating PDE. |
| *k33* | nM-1.s-1 | 0.0029 | k1 for PKA phosphorylating PDE. |
| *k34* | s-1 | 0.4 | rate for phopho-PDE dephosphorylation. |
| *k35* | nM-1.s-1 | 0.005040068544932 | k1 for basal PDE converting cAMP to AMP. |
| *k36* | s-1 | 40 | k2 for basal PDE converting cAMP to AMP. |
| *k37* | s-1 | 0.066125 | k3 for basal PDE converting cAMP to AMP. |
| *k38* | nM-1.s-1 | 0.005040068544932 | k1 for active PDE converting cAMP to AMP. |
| *k39* | s-1 | 50 | k2 for active PDE converting cAMP to AMP. |
| *k40* | s-1 | 50 | k3 for active PDE converting cAMP to AMP. |
| *k41* | s-1 | 0.000035 | rate for basal G-protein activation. |
| *k42* | s-1 | 0.0 | rate for ligand-receptor internalization. |
| *k43* | s-1 | 30 | rate for GTP hydrolysis. |
| *k44* | s-1 | 0.0025 | kf for active PKA nuclear translocation. |
| *k45* | s-1 | 0.00002 | kb for active PKA nuclear translocation. |
| *k46* | nM-1.s-1 | 0.45 | k1 for PKA phosphorylating SF1. |
| *k47* | s-1 | 0.0225 | k2 for PKA phosphorylating SF1. |
| *k48* | s-1 | 450 | k3 for PKA phosphorylating SF1. |
| *k49* | nM-1.s-1 | 0.030 | kf for nuclear inhibitor binding to nuclear PKA. |
| *k50* | s-1 | 0.2 | kb for nuclear inhibitor binding to nuclear PKA. |
| *k51* | s-1 | 0.0005 | kf for inhibited PKA translocation to cytoplasm. |
| *k52* | s-1 | 0.005 | kb for inhibited PKA translocation to cytoplasm. |
| *k53* | nM-1.s-1 | 0.15 | kf for first PKA binding to regulatory dimer. |
| *k54* | nM-1.s-1 | 0.0000004523 | kb for first PKA binding to regulatory dimer. |
| *k55* | nM-1.s-1 | 0.15 | kf for second PKA binding to regulatory dimer. |
| *k56* | nM-1.s-1 | 0.0000000175 | kb for second PKA binding to regulatory dimer. |
| *k57* | s-1 | 0.15 | kf for PKA inhibitor nuclear translocation. |
| *k58* | s-1 | 0.005 | kb for PKA inhibitor nuclear translocation. |
| *k59* | s-1 | 0.05 | SF1P dephosphorylation rate. |
| *k60* | s-1 | 0.0086 | rate for PKA Regulatory unit and cAMP dissociate. |
| *V_spSTAR_* | nMs-1 | 0.00041667 | Maximum rate for StAR mRNA synthesis. |
| *K_cpSTAR_* | nM | 0.7 | Activation Constant for enhancement of StAR |
| *K_mpSTAR_* | nM | 0.31 | Michaelis constant for degradation of StAR mRNA |
| *V_mpSTAR_* | nMs-1 | 0.00061112 | Maximum rate for StAR mRNA degradation. |
| *K_dmpSTAR_* | s-1 | 0.00000277778 | Nonspecific degradation rate constant for mRNA |
| n |  | 2 | Degree of cooperativity of transcription factors |
| V_prSTAR_ | nMs-1 | 0.00816 | Maximum rate for StAR protein synthesis. |
| *K_prSTAR_* | nM | 0.7 | Michaelis Constant for StAR protein synthesis |
| *K_dprSTAR_* | s-1 | 0.0003 | Nonspecific degradation rate constant for StAR protein |
| *V_ch_* | nMs-1 | 0.05 | Maximum rate for cholesterol uptake into the inner mitochondrial membrane |
| *K_ch_* | nM | 0.7 | Michaelis Constant for cholesterol uptake into the inner mitochondrial membrane |
| *V_spLH_* | nMs-1 | 0.00041667 | Maximum rate for LH mRNA synthesis. |
| *K_cpLH_* | nM | 0.7 | Activation Constant for enhancement of LH |
| *K_mpLH_* | nM | 0.31 | Michaelis constant for degradation of LH mRNA |
| *V_mpLH_* | nMs-1 | 0.00061112 | Maximum rate for LH mRNA degradation. |
| *K_dmpLH_* | s-1 | 0.00000277778 | Nonspecific degradation rate constant for LH mRNA |
| n |  | 2 | Degree of cooperativity of transcription factors |
| *V_prLH_* | nMs-1 | 0.00816 | Maximum rate for LH protein synthesis. |
| *K_prLH_* | nM | 0.7 | Michaelis Constant for LH protein synthesis |
| *K_dprLH_* | s-1 | 0.0003 | Nonspecific degradation rate constant for LH protein |
| *K_inLH_* | nM-1s-1 | 0.01 | Inhibition constant of E2 and T on LH mRNa |
| *km1* | s-1 | 0.001 | Metabolite parameters [24] |
| *km2* | s-1 | 0.006 | Metabolite parameters [24] |
| *Km3* | s-1 | 0.004 | Metabolite parameters [24] |
| *Km4* | s-1 | 0.004 | Metabolite parameters [24] |
| *Km5* | s-1 | 0.002 | Metabolite parameters [24] |
| *Km6* | s-1 | 0.004 | Metabolite parameters [24] |
| *Km7* | s-1 | 0.004 | Metabolite parameters [24] |
| *Km8* | s-1 | 0.006 | Metabolite parameters [24] |
| *Km9* | s-1 | 2.5139e-3 | Metabolite parameters [24] |
| *Km10* | s-1 | 0.001 | Metabolite parameters [24] |
| *km11* | s-1 | 0.0016 | Metabolite parameters [24] |
| *Km12* | s-1 | 2.88e-4 | Metabolite parameters [24] |
| *km13* | s-1 | 0.000589 | Metabolite parameters [24] |
| *km14* | s-1 | 0.001 | Metabolite parameters [24] |
| *km15* | Dimension less | 0.0015 | Metabolite parameters [24] |
| *km16* | nM | 36.4667 | Metabolite parameters [24] |
| *km17* | nM | 20.9189 | Metabolite parameters [24] |
| *km18* | s-1 | .001 | Metabolite parameters [24] |
| *km19* | s-1 | 0.001 | Metabolite parameters [24] |
| *Km20* | s-1 | 0.015 | First order diffusion constant of fadrazole |
| *kdh* | s-1 | 0.001 | Degradation rate constant of plasma steroids |
