# Supplementary material for: Fathead minnow steroidogenesis: in silico analyses reveals tradeoffs between nominal target efficacy and robustness to cross-talk
Source: BMC Syst Biol. 2010 Jun 28;4:89. doi: 10.1186/1752-0509-4-89 (PMC2905341; doi:10.1186/1752-0509-4-89)
Supplement: Additional file 5 — Flux balance analysis of ovarian steroidogenesis model. This file describes how Flux Balance Analysis was conducted. [file 1752-0509-4-89-S5.DOC]

# Additional file 5 - Flux balance analysis of ovarian steroidogenesis model.

Flux balance analysis (FBA) quantifies the metabolic network based on steady state approximations with knowledge of the reaction stoichiometry. Mathematically, this can be expressed as


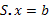


where
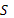
 denotes the stoichiometric matrix of the metabolic reactions,
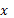
 is the unknown vector of fluxes and
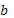
 is known vector of accumulation rates for metabolites. For intracellular metabolites, the values of the
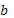
 vector is set to zero and specific values are assigned for extracellular metabolites.

Fig. S1 shows the schematic of the steroidogenesis in vitro model consisting of two components: ovary and medium. Cholesterol is precursor for all steroid hormones and supplied from medium. There are two steroids (testosterone, T and estradiol, E2) that are excreted into the media. Firstly, we examined network analysis with different possible objective functions with normalized cholesterol uptake rate equal to 100. Results show that the flux distribution of the metabolic network in theca cell (except for reaction AD to T) was independent of choice of the objective function and the normalized flux distribution of theca cells is shown. This suggests that the relative activity of these enzymes does not alter during the steroidogenesis process. We observed no change of flux distribution of the metabolic reactions associated with (
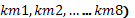
 parameters from FBA analysis. Therefore, we assume that metabolite ratios in the ODE model should be consistent with those calculated using FBA. Further, during data analysis we observed that concentrations of the sex steroids are within 1 – 2 nM. Thus, we fix the range of the parameter values to match our experimental steroid concentrations.


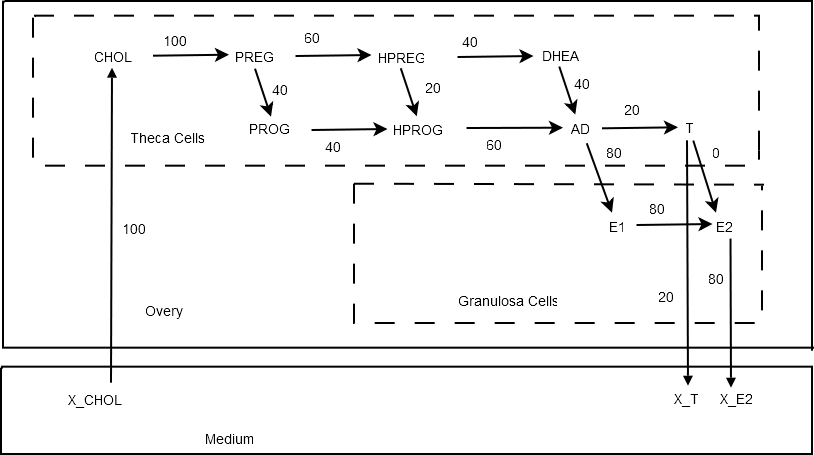


Fig. S1- Flux distribution of the steroidogenesis process for the conceptual model. Decision variables: X_CHOL - 100, and X_T – 20 and T to E2 reaction is set to zero. Objective function was maximization of E2.
