# Supplementary material for: Fathead minnow steroidogenesis: in silico analyses reveals tradeoffs between nominal target efficacy and robustness to cross-talk
Source: BMC Syst Biol. 2010 Jun 28;4:89. doi: 10.1186/1752-0509-4-89 (PMC2905341; doi:10.1186/1752-0509-4-89)
Supplement: Additional file 6 — Initial model conditions. This file describes the initial condition values for each variable within the model. [file 1752-0509-4-89-S6.DOCX]

## Additional file 6 - Initial model conditions

| Species | Conc (nM) |
| --- | --- |
| R | 27.81 |
| LR | 0.00 |
| GDPGasbg | 999.91 |
| RGDPGasbg | 55.61 |
| LRGDPGasbg | 0.00 |
| GTPGas | 0.01 |
| GasAC | 0.00 |
| GDPGas | 448.82 |
| Gbg | 0.01 |
| AC | 12.96 |
| GasACATP | 0.29 |
| cAMP | 0.15 |
| cAMPPDE | 500.08 |
| cAMPPDEa | 0.00 |
| cAMPR2C2 | 0.06 |
| cAMP2R2C2 | 0.00 |
| cAMP3R2C2 | 0.00 |
| cAMP4R2C2 | 0.00 |
| cAMP4R2C | 0.00 |
| cAMP4R2 | 0.00 |
| R2 | 0.00 |
| R2C | 0.18 |
| R2C2 | 498.67 |
| PKAac | 0.00 |
| inPKAc | 0.00 |
| PKAinc | 7.87 |
| cAMPAMPPDEPDEc | 0.01 |
| cAMPPDEaPDEac | 0.00 |
| PKAapPDEc | 0.00 |
| PKAan | 0.00 |
| inPKAn | 0.03 |
| PKAinn | 235.95 |
| SF1 | 79.43 |
| SF1a | 0.57 |
| CREBPKAan | 0.00 |
| StAR mRNA | 0.09 |
| StAR protein | 2.96 |
| Cholesterol outer mitocondrial membrane | 60000.00 |
| Cholesterol inner mitocondrial membrane | 3.99 |
| Inhibin mRHA | 0.01 |
| Inhibin protein | 0.25 |
| LH mRHA | 0.00 |
| LH protein | 0.00 |
| PREG | 0.40 |
| HPREG | 0.40 |
| DHEA | 0.40 |
| PROG | 0.40 |
| HPROG | 0.40 |
| AD | 0.49 |
| T | 1.44 |
| E1 | 0.50 |
| E2 | 1.57 |
| Ex_AD | 4.92 |
| Ex_E1 | 4.95 |
| Ex_T | 14.36 |
| Ex_E2 | 15.68 |
| Ovary FAD concentration | 0.00 |
